# Risk factors for gallstones and kidney stones in a cohort of patients with inflammatory bowel diseases

**DOI:** 10.1371/journal.pone.0185193

**Published:** 2017-10-12

**Authors:** Stefania Fagagnini, Henriette Heinrich, Jean-Benoît Rossel, Luc Biedermann, Pascal Frei, Jonas Zeitz, Marianne Spalinger, Edouard Battegay, Lukas Zimmerli, Stephan R. Vavricka, Gerhard Rogler, Michael Scharl, Benjamin Misselwitz

**Affiliations:** 1 Division of Gastroenterology and Hepatology, University Hospital Zurich (USZ) and Zurich University, Zurich, Switzerland; 2 Division of Internal Medicine, University Hospital Zurich, and Zurich University, Zurich, Switzerland; 3 Health Care Evaluation Unit, Institute of Social and Preventive Medicine, Lausanne, Switzerland; 4 Gastroenterology Bethanien, Zurich, Switzerland; 5 Department of Internal Medicine, Kantonsspital Olten, Olten, Switzerland; 6 Division of Gastroenterology and Hepatology, Stadtspital Triemli, Zurich, Switzerland; 7 Zurich Center for Integrative Human Physiology, University of Zurich, Zurich, Switzerland; University Hospital Llandough, UNITED KINGDOM

## Abstract

**Background:**

Gallstones and kidney stones are known complications of inflammatory bowel diseases (IBD). Risk factors have been insufficiently studied and explanatory studies date back up to 30 years. It remains unclear, whether improved treatment options also influenced risk factors for these complications.

**Objectives:**

Identifying risk factors for gallstones and kidney stones in IBD patients.

**Methods:**

Using data from the Swiss Inflammatory Bowel Disease Cohort Study we assessed associations of diseases characteristics with gallstones and kidney stones in univariate and multivariate logistic regression analyses.

**Results:**

Out of 2323 IBD patients, 104 (7.8%) Crohn’s disease (CD) and 38 (3.8%) ulcerative colitis (UC) patients were diagnosed with gallstones. Significant risk factors for gallstones were diagnosis of CD, age at diagnosis, disease activity and duration, NSAID intake, extra-intestinal manifestations and intestinal surgery. Kidney stones were described in 61 (4.6%) CD and 30 (3.0%) UC patients. Male gender, disease activity, intestinal surgery, NSAID usage and reduced physical activity were significant risk factors. Hospitalization was associated with gallstones and kidney stones. The presence of gallstones increased the risk for kidney stones (OR 4.87, p<0.001).

**Conclusion:**

The diagnosis of CD, intestinal surgery, prolonged NSAID use, disease activity and duration and bowel stenosis were significantly associated with cholecystonephrolithiasis in IBD.

## Introduction

Inflammatory bowel diseases (IBD) comprise chronic inflammatory diseases of the gastrointestinal tract (GIT), including Crohn’s disease (CD), ulcerative colitis (UC) and indeterminate colitis (IC). The diseases are associated with lifelong morbidity and increased mortality [1, 2].

Frequently, patients suffer from extraintestinal complications of IBD. An increased risk of gallstone disease in CD patients is well established [3–9]. A recent meta-analysis concluded that the risk of gallstones in CD was increased by an odds ratio (OR) of 2.05 [10]. In contrast, the risk of gallstones in UC is uncertain. Some studies have suggested an increased risk [11] but no significant difference in prevalence of gallstones could be observed in the recent meta-analysis from Zhang et al. (OR 1.12) [10].

Renal involvement is also considered an extraintestinal complication of IBD and typical manifestations include nephrolithiasis, tubulointerstitial nephritis, glomerulonephritis and amyloidosis [12]. Kidney stones may arise from chronic inflammation, changes in intestinal physiology due to inflammation, surgery or intestinal malabsorption. The reported frequency of nephrolithiasis ranges from 0.2%-40% in patients with IBD and intestinal surgery was consistently reported as a strong risk factor [13–17].

However, patient data for gallstones and kidney stones are limited and studies were insufficiently powered to detect potentially relevant risk factors. In addition, many relevant studies date back up to 50 years [15, 18]. Considering dramatic changes in clinical practice within the last 20 years, it is not clear, which risk factors apply for our patients today.

The identification of risk factors for gallstones and nephrolithiasis remains an important task to prevent complications and identify patients who would benefit from potentially protective interventions. We therefore aimed to assess risk factors using data from the Swiss IBD cohort study, a large prospective cohort of well-characterized patients.

We provide a comprehensive analysis of risk factors for gallstones and kidney stones, potentially enabling prophylactic interventions.

## Materials and methods

### Patient data

Data were retrieved from the nationwide Swiss Inflammatory Bowel Disease Cohort Study (SIBDCS). The SIBDCS is a multicenter prospective observational population-based study and includes patients with IBD from Switzerland. The study was extended to all regions of Switzerland in 2006 in a multidisciplinary effort by gastroenterologists, pathologists, psychologists and bioinformatics specialists. The cohort study is funded by the Swiss National Science Foundation (SNF). For inclusion in the study, all patients must have a diagnosis established at least 4 months prior to inclusion. Data are prospectively collected once a year and entered into a central database [19].

### Study design

Using the exclusive patient collective of the SIBDCS we evaluated complications in IBD patients and assessed potential associations with specific diseases characteristics. Univariate and multivariate logistic regression analyses were performed, each time with a complication as response. The following possible explanatory variables were considered: 1) Epidemiological characteristics: diagnosis of CD or UC, disease duration (years), gender, age at diagnosis and body mass index (BMI), physical activity. 77 patients with indeterminate colitis (IC) were excluded. If not indicated otherwise, patients with CD were considered as a single entity and patients with Crohn’s colitis and Crohn’s ileitis were not distinguished. 2) Disease characteristics: activity index, disease location, extra-intestinal manifestations (EIM), existence of stenosis, fistula, fissure and abscess, intestinal surgery. 3) Selected co-medications: steroids, non-steroidal anti-inflammatory drugs (NSAID).

To assess disease activity and allow comparison between UC (Modified Truelove and Witts activity index, MTWAI) and CD (Crohn’s disease activity index, CDAI), disease activity measures were normalized to a value between 0 and 100 and expressed as an activity index. For a diagnosis of gallstones, or kidney stones all appropriate diagnostic modalities including abdominal ultrasound or CT scan, MRI, X-ray or surgical examination upon cholecystectomy would be considered. The cohort design did not permit assessment of exact imaging modalities leading to diagnosis and symptom status at diagnosis.

Whenever possible, the disease characteristics immediately preceding the diagnosis of gallstones were used. Patients with missing values in relevant variables were excluded.

For a secondary analysis, we performed univariate and multivariate logistic regressions with gallstones or nephrolithiasis as response and specific surgical procedures as explanatory variables. Finally, we tested for a dependency between hospitalization and the existence of gallstones/kidney stones.

### Statistical analysis

In order to build a multivariate logistic regression model, we first performed univariate regressions with each factor mentioned in Table 1. We then fit together all variables such that the corresponding p-value in univariate regressions was less than 0.2. In the presence of certain variables, others may cease to be important. The multivariate model was then built by removing nonsignificant covariates one after each other, based on likelihood ratio tests. We then considered again each factor mentioned in Table 1 and tried to include them in the model subsequently. We finally checked that no factor in the model could be removed and that no factor could be added into the model, always based on likelihood ratio tests. For all analyses we calculated adjusted odds ratios (OR) with 95% confidence intervals. A p-value of less than 0.05 was considered statistically significant. For this analysis Stata software was used (StataCorp. 2015. Stata Statistical Software: Release 14. College Station, TX: StatCorp LP).

**Table 1:** Explanatory variable in IBD patients with complications.

	Last visit before Gallstones ^a^		Last visit before Nephrolithiasis	
	CD	UC	CD	UC
**Number of patients**	1333 (57.4%)	990 (42.6%)	1333 (57.4%)	990 (42.6%)
**Males**	606 (45.5)	521 (52.6)	606 (45.5%)	521 (52.7%)
**Gallstones**	104 (7.8)	38 (3.8)		
**Nephrolithiasis**			61 (4.6%)	30 (3.0%)
**NSAID ^b^**				
**No**	1063 (79.7)	824 (83.2)	1063 (79.7)	824 (83.2)
**Yes**	211 (15.8)	110 (11.1)	211 (15.8)	110 (11.1)
**Unknown**	59 (4.4)	56 (5.7)	59 (4.4)	56 (5.7)
**EIM**				
**No**	594 (44.6)	564 (57.0)	583 (43.7)	560 (56.6)
**Yes**	739 (55.4)	426 (43.0)	750 (56.3)	430 (43.4)
**Fistula**				
**No**	711 (53.3)		709 (53.2)	
**Yes**	622 (46.7)		624 (46.8)	
**Stenosis**				
**No**	753 (56.5)		752 (56.4)	
**Yes**	580 (43.5)		581 (43.6)	
**Intestinal Surgery**				
**No**	772 (57.9)	891 (90.0)	771 (57.8)	891 (90.0)
**Yes**	561 (42.1)	99 (10.0)	562 (42.2)	99 (10.0)
**Therapy with Steroids**				
**No**	195 (14.6)	204 (20.6)	195 (14.6)	204 (20.6)
**Yes**	1138 (85.4)	786 (79.4)	1138 (85.4)	786 (79.4)
**Last CD location**				
**L1 (ileal)**	399 (29.9)		388 (29.1)	
**L2 (colonic)**	441 (33.1)		446 (33.5)	
**L3 (ileo-colonic)**	417 (31.3)		418 (31.4)	
**L4 (upper GI only)** **Unknown**	33 (2.5) 43 (3.2)		35 (2.6) 46 (3.4)	
**Last UC/IC location**				
**Proctitis**		234 (23.6)		233 (23.5)
**Left-sided colitis**		375 (37.9)		378 (38.2)
**Pancolitis**		364 (36.8)		362 (36.6)
**Unknown**		17 (1.7)		17 (1.7)
**Age at Diagnosis**				
**[median,** **q25 –q75,** **min–max]**	[26.2, 20.1–36.5, 2.9–81.4], n = 1331	[30.9, 23.3–40.3, 3.4–82.2], n = 987	[26.2, 20.1–36.5, 2.9–81.4], n = 1331	[30.9, 23.3–40.3, 3.4–82.2], n = 987
**Disease duration**				
**[median,** **q25 –q75,** **min–max]**	[12.7, 6.8–21.5, 0.1–52.5], n = 1331	[11.3, 6.2–18.2, 0.1–52.4], n = 987	[12.8, 6.9–21.9, 0.1–56.6], n = 1331	[11.2, 6.2–18.2, 0.1–49.1], n = 987
**Last BMI**				
**[median,** **q25 –q75,** **min–max]**	[23.5, 21.1–26.5, 15.4–48.3], n = 1296	[24.2, 21.7–26.9, 16.3–50.4], n = 968	[23.5, 21.1–26.5, 15.4–48.3], n = 1296	[24.2, 21.7–26.9, 16.3–46.3], n = 968
**Last CDAI**				
**[median,** **q25 –q75,** **min–max]**	[25, 6–55, 0–450], n = 1333		[25, 6–57, 0–435], n = 1333	
**Last MTWAI** **[median,**		[2,		[2,
**q25 –q75,** **min–max]**		0–4, 0–16], n = 990		0–4, 0–16], n = 990
**Physical activity ^b^**				
**Never**	400 (30.0)	239 (24.1)	400 (30.0)	239 (24.1)
**Monthly**	528 (39.6)	379 (38.3)	528 (39.6)	379 (38.3)
**Weekly or daily**	405 (30.4)	372 (37.6)	400 (30.0)	372 (37.6)

a) Patients with cholecystectomy are included in the gallstone group.

b) NSAID intake and physical activity are only mentioned in enrollment questionnaire.

CD: Crohn`s disease; UC: Ulcerative colitis; NSAID: Non-steroidal anti-inflammatory drugs; EIM: Extraintestinal manifestation; CDAI: Crohn`s disease activity index; MTWAI: Modified Truelove and Witts activity index

### Ethical considerations

The IBD cohort study has been approved by all ethics committees in Switzerland (leading Ethics Committee: Ethics Committee of the Canton Zürich, approval number EK-1316) All patients signed an informed consent and confirmed their participation in the cohort study at the time of enrollment and gave informed consent for data collection and analysis for research purposes. The current substudy has been evaluated and approved by the scientific board of SIBDCS.

## Results

For our analysis a group of 2323 IBD patients was considered of whom 1333 (55.4%) suffered from CD and 999 (42.6%) from UC. Basic epidemiological variables and distribution of possible risk factors for complications are presented in Table 1. Our cohort reveals the typical distribution of disease parameters of a large IBD cohort, spanning the whole range of disease characteristics from mild to severe disease.

### Risk factors for gallstones in IBD patients

Gallstones were reported in 104 patients with CD (7.8%) and 38 patients with UC (3.8%). In the univariate analysis the diagnosis of CD was significantly associated with gallstones. Higher disease activity, long disease duration, existence of stenosis, intestinal surgery and usage of NSAID, as well as epidemiological characteristics like age, were also significantly related to the presence of gallstones (Table 2).

**Table 2:** Univariate analysis of risk factors for gallstones and nephrolithiasis.

UNIVARIATE LOGISTIC REGRESSIONS	Gallstones OR (95% CI; p-value)	Kidney stones OR (95% CI; p-value)
**Diagnosis**		
CD	1 (ref)	1 (ref)
UC	0.47 (0.32–0.69; **< 0.001**)	0.65 (0.42–1.02; 0.06)
**Gender**		
Men	1 (ref)	1 (ref)
Women	0.97 (0.69–1.36; 0.85)	0.58 (0.37–0.89; **0.01**)
**BMI**	1.02 (0.98–1.06; 0.38)	0.98 (0.93–1.03; 0.41)
**Steroids at baseline**		
No	1 (ref)	1 (ref)
Yes	1.02 (0.65–1.61; 0.93)	1.38 (0.75–2.56; 0.31)
**NSAID intake (at least once)**		
No	1 (ref)	1 (ref)
Yes	1.68 (1.10–2.57; **0.02**)	2.27 (1.40–3.68; **0.001**)
**Existence of stenosis**		
No	1 (ref)	1 (ref)
Yes	2.44 (1.73–3.44; **< 0.001**)	1.82 (1.18–2.80; **0.007**)
**Fistula, fissure or abscess**		
No	1 (ref)	1 (ref)
Yes	1.37 (0.96–1.95; 0.09)	2.01 (1.32–3.07; **0.001**)
**Intestinal surgery**		
No	1 (ref)	1 (ref)
Yes	3.79 (2.68–5.36; **< 0.001**)	2.95 (1.93–4.50; **< 0.001**)
**EIM**		
No	1 (ref)	1 (ref)
Yes	0.94 (0.67–1.31; 0.70)	1.25 (0.82–1.90; 0.31)
**Age at diagnosis**	1.01 (1.00–1.02; 0.05)	1.00 (0.98–1.02; 0.96)
**Disease Duration**	1.03 (1.02–1.05; **< 0.001**)	1.03 (1.01–1.05; **0.001**)
**Last Activity Index ^a^**	1.03 (1.02–1.04; **< 0.001**)	1.04 (1.02–1.05; **< 0.001**)
**Physical activity** Never	1 (ref)	1 (ref)
Monthly	0.78 (0.52–1.17; 0.23)	0.57 (0.35–0.91; **0.02**)
Weekly or daily	0.67 (0.43–1.03; 0.07)	0.36 (0.20–0.63; **< 0.001**)

a) Number between 0 and 100 which is obtained by dividing the CDAI score by 5 and the MTWAI score by 21 and then multiply it by 100.

CD: Crohn`s disease; UC: Ulcerative colitis; NSAID: Non-steroidal anti-inflammatory drugs; EIM: Extraintestinal manifestation; CDAI: Crohn`s disease activity index; MTWAI: Modified Truelove and Witts activity index

Multivariate logistic regression analysis confirmed the association of seven risk factors with gallstones: diagnosis of CD, age at diagnosis, disease duration, intestinal surgery, EIM, NSAID intake and disease activity (Table 3). For disease duration, we calculated an OR of 1.026 per year, resulting in a cumulative risk of 1.7 (1.026^20) in 20 years. For disease activity an OR of 1.037 per point of our activity index was calculated, resulting in an OR of 18.3 for an individual with a CDAI of 500 compared to a CDAI of 100.

**Table 3:** Multivariate analysis of risk factors regarding gallstones.

MULTIVARIATE LOGISTIC REGRESSION	Odds Ratio (95% CI; p-value)
(Gallstones, all patients, n = 2203)	
**Diagnosis**	
CD	1 (ref)
UC	0.517 (0.329–0.814; **0.004**)
**Age at Diagnosis (per year)**	1.023 (1.009–1.036; **0.001**)
**Disease Duration (per year)**	1.026 (1.008–1.045; **0.005**)
**Intestinal Surgery**	
No	1 (ref)
Yes	2.623 (1.734–3.968; **< 0.001**)
**EIM**	
No	1 (ref)
Yes	0.546 (0.372–0.801; **0.002**)
**NSAID intake**	
No	1 (ref)
Yes	1.715 (1.087–2.708; **0.021**)
**Activity Index (per point)**	1.037 (1.025–1.050; **< 0.001**)

CD: Crohn`s disease; UC: Ulcerative colitis; NSAID: Non-steroidal anti-inflammatory drugs; EIM: Extraintestinal manifestation

When patients with CD and UC were considered separately (S1 and S2 Tables), disease activity and intestinal surgery remained significant risk factors gallstones in both, CD and UC. NSAID usage was a specific risk factor for gallstones in UC but not in CD. Disease location in CD was an important risk factor for gallstones with significantly higher risk for gallstones in patients with ileal involvement. (S1 Table)

### Risk factors for nephrolithiasis in IBD patients

Kidney stones were reported in 92 patients, 61 patients with CD (4.6%) and 30 patients with UC (3.0%). According to univariate regression analysis (Table 2) a diagnosis of CD and disease phenotypes (bowel stenosis, perianal disease) were risk factors for nephrolithiasis. Disease duration and activity as well as intestinal surgery, intake of NSAID, male gender and deficiency in physical activity (less than once a week) were also associated with kidney stones.

Using all variables for calculation of a multivariate logistic regression model, five risk factors (male gender, intestinal surgery and NSAID intake, disease activity and deficiency in physical activity) remained significant (Table 4).

**Table 4:** Multivariate analysis of risk factors regarding nephrolithiasis.

MULTIVARIATE LOGISTIC REGRESSION	Odds Ratio (95% CI; p-value)
(Kidney stones, all patients, n = 2208)	
**Gender**	
Men	1 (ref)
Women	0.533 (0.341–0.833; **0.006**)
**Intestinal Surgery**	
No	1 (ref)
Yes	2.461 (1.591–3.805; **< 0.001**)
**NSAID intake**	
No	1 (ref)
Yes	2.334 (1.415–3.851; **0.001**)
**Activity Index**	1.032 (1.018–1.045; **< 0.001**)
**Physical activity**	
Never	1 (ref)
Monthly	0.699 (0.426–1.146; 0.156)
Weekly or Daily	0.434 (0.242–0.780; **0.005**)

NSAID: Non-steroidal anti-inflammatory drugs

When patients with CD and UC were considered separately, male gender and disease activity were significant risk factors in both diseases. In CD patients, intestinal surgery and physical activity were additional risk factors; in UC patients NSAID usage was a predictive factor for nephrolithiasis (S3 and S4 Tables).

### Surgical procedures and gallstones in patients with CD and UC

**Fig 1:**
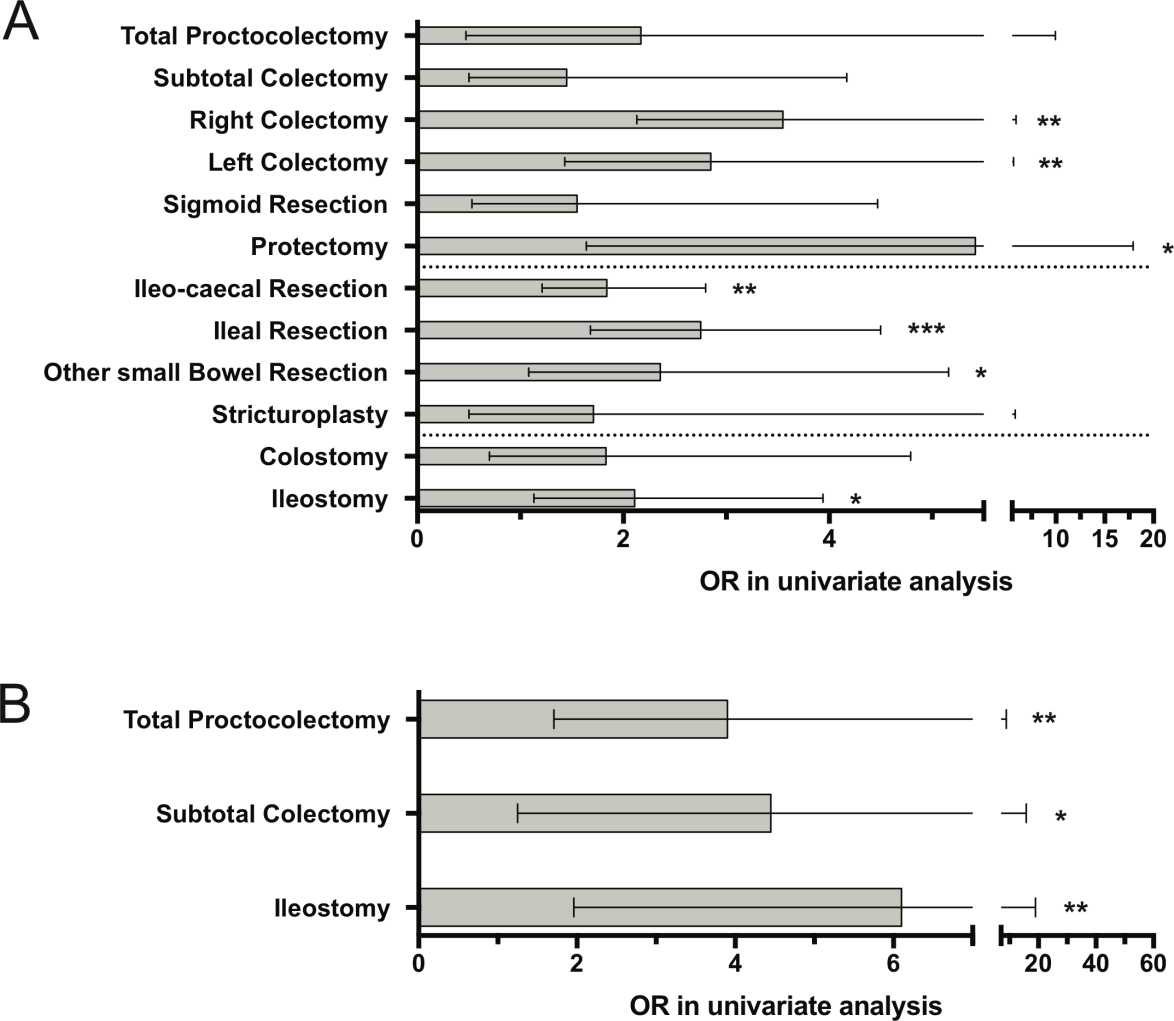
Past intestinal surgery and the risk of gallstones in A: CD patients and B: UC patients. The odds ratio (OR) compared to the whole study population is indicated. * p<0.05, ** p<0.001; *** p<0.0001.

Intestinal surgery was performed in 561 patients with CD and 99 UC patients (Fig 1). In CD, ileal and ileo-caecal resection as well as ileostomy, total proctocolectomy, colectomy (right and left) and proctectomy significantly increased the risk for gallstone disease. In contrast, other surgical procedures, such as sigmoid resection or stricturoplasty were not associated with gallstones. In UC were total proctocolectomy and ileostomy were significant risk factors (Fig 1).

After adjustment for risk factors from the multivariate model (last diagnosis, existence of surgery, age at diagnosis, disease duration, last activity index, existence of EIM and intake of NSAID), right colectomy was a significant risk in CD patients (OR 1.8, 95% CI: 1.037–3.420, p = 0.038).

### Surgical procedures and kidney stones in patients with CD and UC/

**Fig 2:**
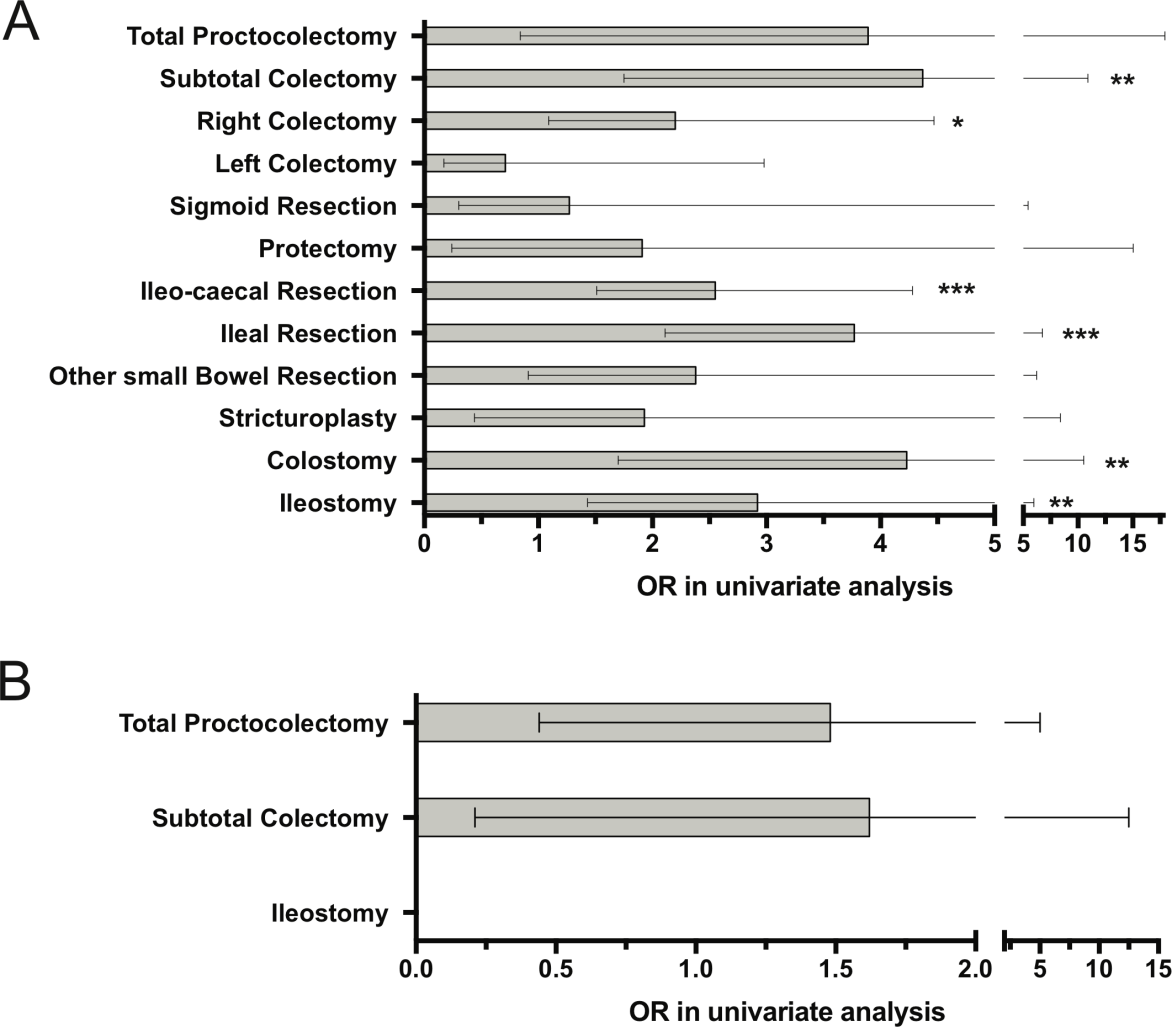
Past intestinal surgery and the risk of kidney stones in A: CD patients and B: UC patients. The odds ratio (OR) compared to the whole study population is indicated. * p<0.05, ** p<0.001; *** p<0.0001.

In CD patients, nephrolithiasis was associated with surgery of the small intestine, subtotal and right colectomy and with colostomy and ileostomy (Fig 2). After adjustment for risk factors from the multivariate model (gender, NSAID intake, existence of surgery, last activity index, physical activity), colostomy stayed a significant risk factor (OR 3.0, CI: 1.1–7.9, p = 0.028). In UC patients no individual kind of surgery was significant.

### Association of gallstones and nephrolithiasis

The occurrence of gallstones and kidney stones were significantly correlated. The presence of gallstones or kidney stones increased the risk for the other complication by an OR of 4.87, 95% CI: 2.8–8.0, p<0.001).

### Hospitalization as a predictor for gallstones and kidney stones

Hospitalization was a risk factor for gallstones: 52 out of 148 patients (35.1%) with a hospitalization during the last 12 months vs. 96 out of 2323 patients (4.1%) without a hospitalization had a diagnosis of gallstones (OR 12.6; CI: 8.5–18.7, p<0.001) (S5 Table). This effect remained stable in the subgroup of patients with CD and UC (OR 9.80 and 20.15, respectively; p<0.001.

Kidney stones were also more prevalent in patients with a previous hospitalization and 36 out of 136 patients (26.5%) with a hospitalization vs. 55 out of 2132 patients (2.5%) without a previous hospitalization had a diagnosis of kidney stones (OR 13.9; CI: 8.8–22.1, p<0.001) (S6 Table). This difference remained robust in the subgroup of patients with CD and UC (OR 13.3 and 14.4, p<0.001).

## Discussion

We provide a comprehensive analysis of risk factors for gallstones and kidney stones in a large cohort of Swiss IBD patients. Gallstones were associated with diagnosis of CD, age at diagnosis, disease duration and activity, past intestinal surgery, EIM and NSAID intake with strong statistical significance. Kidney stones shared most of these risk factors including disease activity, intestinal surgery and NSAID intake but also showed association with male gender and reduced physical activity.

In the literature there is general agreement that CD patients have an increased risk of gallstones [3–10]. Past intestinal surgery [4, 6, 7, 9] and age [4, 6–8] are well-established risk factors, while disease duration was confirmed by some [4] but excluded by other studies [8]. Disease activity was strongly associated with gallstones in our study but had not been considered in previous work.

561 out of 1339 CD patients underwent intestinal surgery and detailed information regarding surgical procedures was available. Ileostomy was associated with a strong risk for gallstones. Most major surgical procedures of the colon and the ileum were also associated with gallstones while other smaller procedures (stricturoplasty) were not. Past work demonstrated length of the resected small intestine [9] and the number of procedures [5, 6] as risk factors. We also observe a dependency between a recent hospitalization (within the last 12 months) and the existence of gallstones as described before [9].

In contrast to CD, less information for UC and gallstone is available. While a recent meta-analysis concluded that the risk of gallstones for UC was not significantly elevated (OR 1.12, CI 0.75–1.68), a few single studies demonstrated an increased risk for gallstones in UC [11, 20] with an OR up to 3.6 [21]. The only identified risk factor was pancolitis [11]. Significant association of disease activity as described in our study argues for a specific risk of gallstones in UC. Surgery, especially right colectomy, total proctocolectomy and ileostomy will increase this risk further.

Several mechanisms explaining an increased risk for gallstones in IBD have been proposed: i) Bile acid malabsorption in enterohepatic circulation after ileal disease or resection would lead to cholesterol supersaturated bile [22, 23]. However, this mechanism remains controversial since changes in bile acid saturation in patients with ileal disease or resection were shown to be transient and several studies reported even a lower cholesterol concentration in bile in patients with CD than healthy subjects [24, 25]. ii) In CD patients without of a functional ileum, higher bilirubin levels were observed in bile, potentially resulting in pigment stones [26]. The authors speculate that in these patients, decreased bile acid absorption would result in higher bilirubin solubilisation in the colon and increased bilirubin reabsorption. iii) Additional mechanisms for gallstones in IBD patients include prolonged fasting state, parenteral nutrition and decreased gall bladder motility [27, 28]. One study concluded that intestinal surgery itself, not the altered anatomy after surgery would increase the risk of gallstones [4]. Our analysis remains sufficiently powered to analyse a range of risk factors. Our results argue for several not mutually exclusive disease mechanisms. Disease activity and duration affected the risk for gallstones in all IBD patients. Both parameters would be associated with fasting and bowel rest. In contrast, intestinal surgery and diagnosis of CD point to an altered intestinal physiology as a mechanism for gallstones.

Nephrolithiasis results from urine supersaturation of some components including oxalate or urate. Therefore, low urine volume, low urine pH (resulting in urate precipitation), low urine citrate or magnesium concentration (which can act as inhibitors of stone formation) and high urine oxalate levels increase the risk for kidney stones [29]. Various pathophysiological mechanism for nephrolithiasis in IBD-patients are discussed [30]: i) Loss of water and salt in patients with ileostomy or pronounced diarrhoea will lead to more concentrated urine [31]. ii) Due to intestinal malabsorption, patients have less urinary excretion of citrate and magnesium which can act as inhibitors of oxalate stone formation [32]. iii) Due to intestinal malfunction unabsorbed fatty acids bind intraluminal calcium. Therefore, less insoluble calcium oxalate is excreted in the stool, resulting in higher oxalate reabsorption and higher oxalate concentration in the urine. iv) In patients with ileostomies large amounts of alkaline fluids will be lost and the urine of those patients will be acidic. v) Decolonization of the gastrointestinal tract of the oxalate fermenting bacterium *Oxalobacter fromigenes* was associated with hyperoxaluria and kidney stones in IBD patients [33].

Risk factors for kidney stones in the general population include: male gender, high BMI, diabetes mellitus, gout and low socioeconomic status [34]. However, different risk factors were detected in IBD patients: In a study of 218 UC patients with ileal pouch-anal anastomosis (IPAA) low serum bicarbonate level, presence of extraintestinal IBD manifestations and absence of antibiotic use was associated with a higher risk of kidney stones [35]. Another study identified ileocolonic L3 location (CD), high disease activity (UC) and usage of various drugs (ciprofloxacin, steroids, immunomodulators, metronidazole, methotrexate) as risk factors for nephrolithiasis [17].

In our study, intestinal surgery increases the incidence for kidney stones. We provide an estimated risk of various surgical procedures with the highest risk attributable to ileo/ileo-caecal resection in CD (Fig 2). In Knudsen et al. ileostomy per se increases the risk of nephrolithiasis [14], while other studies described surgery with resection of longer segments of the small intestine (50-100cm) as risk factors for kidney stones [36].

NSAID as a standard medication for symptomatic kidney stones [37] have not been described as a strong risk factor for nephrolithiasis. However, animal data demonstrate rapid formation of oxalate stones after application of Cox-2 inhibitors and a high oxalate diet [38]. Therefore, NSAID-mediated fluid retention might lead to stone formation in the presence of a high urinary oxalate concentration but not in other situations. Alternatively, NSAID usage might mark patients incompliant to treatment recommendations or simply indicate treatment for kidney stones.

Our finding of lack of physical activity as a risk factor regarding kidney stones is interesting since a sedentary life style has also been described as a risk factor in some [39] but not all large epidemiological studies [40].

Our data show an overlap of the risk of gallstones and kidney stones. This is in line with data from large epidemiological studies. In multivariate analyses accounting for risk factors such as age, diet and BMI an OR for kidney stones of patients with gallstones was 1.61–1.85 and vice versa the risk of gallstones in patients with a history of kidney stones was 1.17–1.51 [41].

Current guidelines do not recommend specific preventive measures regarding nephrolithiasis or gallstones. However, identification of high-risk patients for gallstones and kidney stones seems to be important in prevention. Strategies to lower the risk of kidney stones include a low oxalate diet, substitution of medium chain fatty acids and an increase in fluid intake [30]. Patients with risk factors for gallstones could be treated with ursodeoxycholic acid. Obviously, these interventions would need to be tested in prospective trials.

Strengths of our study include the large number of patients and the high level of detailed information available for each patient allowing for testing and controlling several potential risk factors for all complications. Important limitations of our study include: i) We did not systematically test all individuals included in the SWISS IBD cohort for gallstones or kidney stones by ultrasound screening. This represents considerable bias, as several clinically silent cases would have been missed and the frequency of gallstones and kidney stones in our cohort is lower than in other IBD cohorts. ii) Because of a lack of a control group of individuals without IBD the absolute risk for gallstones and kidney stones attributable to IBD remains unknown. iii) Our study is not strictly population based. The Swiss IBD cohort study collects data from patients followed in private practice as well as in large hospitals; however, tertiary care centres are slightly over-represented, potentially affecting our conclusions. iv) Complications and symptoms of gallstones and kidney stones were not recorded in our database. Therefore, we cannot distinguish between incidental observations and relevant conditions for both diseases.

In conclusion, our study identifies and confirms specific risk factors in IBD patients for gallstones and kidney stones. Most of these parameters are related to severe disease of long duration as well as intestinal surgery. Our study thus points out characteristics of high-risk patients, which might benefit from preventive strategies.

## Supporting information

S1 STROBE Checklist (DOC)

S1 Table Multivariate analysis of risk factors for gallstones in CD patients. CD: Crohn`s disease; EIM: Extraintestinal manifestation; CDAI: Crohn`s disease activity index.
(DOCX)

S2 Table Multivariate analysis of risk factors for gallstone disease considering UC patients only. UC: Ulcerative colitis; NSAID: Non-steroidal anti-inflammatory drugs; MTWAI: Modified truelove and witts activity index.
(DOCX)

S3 Table Multivariate analysis of risk factors for kidney stones considering CD patients only. CD: Crohn`s disease; CDAI: Crohn`s disease activity index.
(DOCX)

S4 Table Multivariate analysis of risk factors for kidney stones considering UC patients only. UC: Ulcerative colitis; NSAID: Non-steroidal anti-inflammatory drugs; MTWAI: Modified truelove and witts activity index.
(DOCX)

S5 Table Gallstones and previous hospitalization in the last 12 months in all patients. OR: 12.63 (95% CI: 8.54–18.67; p < 0.001); Subgroup analysis; CD patients: OR 9.85 (95% CI: 6.11–15.87; p < 0.001); UC patients: OR 19.42 (95% CI: 9.75–38.69; p < 0.001).
(DOCX)

S6 Table Kidney stones and previous hospitalization in the last 12 months in all patients. OR: 13.90 (95% CI: 8.75–22.10; p < 0.001). Subgroup analysis; CD patients: OR 13.40 (95% CI: 7.58–23.69; p < 0.001); UC patients: OR 13.96 (95% CI: 6.26–31.14; p < 0.001)
(DOCX)

## Abbreviations

CD: Crohn`s disease
UC: Ulcerative colitis
IC: Indeterminate colitis
NSAID: Non-steroidal anti-inflammatory drugs
EIM: Extraintestinal manifestation
BMI: body mass index
CDAI: Crohn`s disease activity index
MTWAI: Modified Truelove and Witts activity index
SIBDCS: Swiss Inflammatory Bowel Disease Cohort Study
