# Supplementary material for: Risk factors for gallstones and kidney stones in a cohort of patients with inflammatory bowel diseases
Source: PLoS One. 2017 Oct 12;12(10):e0185193. doi: 10.1371/journal.pone.0185193 (PMC5638235; doi:10.1371/journal.pone.0185193)
Supplement: S1 Table — CD: Crohn`s disease; EIM: Extraintestinal manifestation; CDAI: Crohn`s disease activity index. (DOCX) [file pone.0185193.s002.docx]

| MULTIVARIATE LOGISTIC REGRESSION  (Gallstones, CD patients, n=1288*) | Odds Ratio (95% CI; p-value) |
| --- | --- |
| Age at Diagnosis  Disease Duration  Intestinal Surgery  No  Yes  EIM  No  Yes  Last CDAI  Last disease location  L1 (ileal)  L2 (colonic)  L3 (ileo-colonic)  L4 (upper GI) | 1.026 (1.010 – 1.043; **0.001**)  1.038 (1.016 – 1.061; **0.001**)  1 (ref)  2.272 (1.376 – 3.751; **0.001**)  1 (ref)  0.454 (0.285 – 0.722; **0.001**)  1.008 (1.005 – 1.012; **< 0.001**)  1 (ref)  0.567 (0.326 – 0.986; **0.044**)  0.616 (0.364 – 1.044; 0.072)  1.973 (0.713 – 5.459; 0.191) |

**Table S1:** Multivariate analysis of risk factors for gallstones in CD patients

CD: Crohn`s disease; EIM: Extraintestinal manifestation; CDAI: Crohn`s disease activity index

45 patients were excluded from analysis as disease location (n=43) and duration was unknown (n=2)
