# Supplementary material for: Risk factors for gallstones and kidney stones in a cohort of patients with inflammatory bowel diseases
Source: PLoS One. 2017 Oct 12;12(10):e0185193. doi: 10.1371/journal.pone.0185193 (PMC5638235; doi:10.1371/journal.pone.0185193)
Supplement: S2 Table — UC: Ulcerative colitis; NSAID: Non-steroidal anti-inflammatory drugs; MTWAI: Modified truelove and witts activity index. (DOCX) [file pone.0185193.s003.docx]

| MULTIVARIATE LOGISTIC REGRESSION  (Gallstones, UC patients, n=934 *) | Odds Ratio (95% CI; p-value) |
| --- | --- |
| Intestinal Surgery  No  Yes  NSAID intake  No  Yes  Last MTWAI | 1 (ref)  2.831 (1.288 – 6.226; 0.010)  1 (ref)  4.119 (1.973 – 8.602; < 0.001)  1.142 (1.044 – 1.250; 0.004) |

**Table S2:** Multivariate analysis of risk factors for gallstone disease considering UC patients only

UC: Ulcerative colitis; NSAID: Non-steroidal anti-inflammatory drugs; MTWAI: Modified truelove and witts activity index

* 56 patients were excluded from analysis due to missing information on NSAID intake
