# Supplementary material for: Risk factors for gallstones and kidney stones in a cohort of patients with inflammatory bowel diseases
Source: PLoS One. 2017 Oct 12;12(10):e0185193. doi: 10.1371/journal.pone.0185193 (PMC5638235; doi:10.1371/journal.pone.0185193)
Supplement: S3 Table — CD: Crohn`s disease; CDAI: Crohn`s disease activity index. (DOCX) [file pone.0185193.s004.docx]

| MULTIVARIATE LOGISTIC REGRESSION  (Kidney stones, CD patients, n=1333) | Odds Ratio (95% CI; p-value) |
| --- | --- |
| Gender  Men  Women  Intestinal Surgery  No  Yes  Last CDAI  Physical activity  Never  Monthly  Weekly or Daily | 1 (ref)  0.570 (0.331 – 0.981; **0.042**)  1 (ref)  3.286 (1.807 – 5.975; **< 0.001**)  1.009 (1.006 – 1.013; **< 0.001**)  1 (ref)  0.530 (0.287 – 0.978; **0.042**)  0.408 (0.197 – 0.843; **0.015**) |

**Table S3:** Multivariate analysis of risk factors for kidney stones considering CD patients only

CD: Crohn`s disease; CDAI: Crohn`s disease activity index
