# Supplementary material for: Risk factors for gallstones and kidney stones in a cohort of patients with inflammatory bowel diseases
Source: PLoS One. 2017 Oct 12;12(10):e0185193. doi: 10.1371/journal.pone.0185193 (PMC5638235; doi:10.1371/journal.pone.0185193)
Supplement: S4 Table — UC: Ulcerative colitis; NSAID: Non-steroidal anti-inflammatory drugs; MTWAI: Modified truelove and witts activity index. (DOCX) [file pone.0185193.s005.docx]

| MULTIVARIATE LOGISTIC REGRESSION  (Kidney stones, UC patients, n=934*) | Odds Ratio (95% CI; p-value) |
| --- | --- |
| Gender  Men  Women  NSAID intake  No  Yes  Last MTWAI | 1 (ref)  0.450 (0.202 – 1.005; 0.051)  1 (ref)  4.879 (2.233 – 10.660; **< 0.001**)  1.119 (1.011 – 1.240; **0.030**) |

**Table S4:** Multivariate analysis of risk factors for kidney stones considering UC patients only

UC: Ulcerative colitis; NSAID: Non-steroidal anti-inflammatory drugs; MTWAI: Modified Truelove and Witts activity index

* 56 patients were excluded from analysis due to missing information on NSAID intake
