# Supplementary material for: Risk factors for gallstones and kidney stones in a cohort of patients with inflammatory bowel diseases
Source: PLoS One. 2017 Oct 12;12(10):e0185193. doi: 10.1371/journal.pone.0185193 (PMC5638235; doi:10.1371/journal.pone.0185193)
Supplement: S5 Table — OR: 12.63 (95% CI: 8.54–18.67; p < 0.001); Subgroup analysis; CD patients: OR 9.85 (95% CI: 6.11–15.87; p < 0.001); UC patients: OR 19.42 (95% CI: 9.75–38.69; p < 0.001). (DOCX) [file pone.0185193.s006.docx]

| ALL PATIENTS | Gallstones: No | Gallstones: Yes | Total |
| --- | --- | --- | --- |
| Previous hospitalizations in the last 12 months: No | 2085 (95.9%) | 90 (4.1%) | 2175 (100%) |
| Previous hospitalizations in the last 12 months: Yes | 96 (64.9%) | 52 (35.1%) | 148 (100%) |
| Total | 2181 (93.9%) | 142 (6.1%) | 2323 (100%) |

**Table S5:** gallstones and previous hospitalization in the last 12 months in all patients

OR: 12.55 (95% CI: 8.53 – 18.68; p < 0.001);

Subgroup analysis; CD patients: OR 9.80 (95% CI: 6.08 – 15.79; p < 0.001); UC patients:

OR 20.25 (95% CI: 9.74 – 42.10; p < 0.001)
