# Supplementary material for: Risk factors for gallstones and kidney stones in a cohort of patients with inflammatory bowel diseases
Source: PLoS One. 2017 Oct 12;12(10):e0185193. doi: 10.1371/journal.pone.0185193 (PMC5638235; doi:10.1371/journal.pone.0185193)
Supplement: S6 Table — OR: 13.90 (95% CI: 8.75–22.10; p < 0.001). Subgroup analysis; CD patients: OR 13.40 (95% CI: 7.58–23.69; p < 0.001); UC patients: OR 13.96 (95% CI: 6.26–31.14; p < 0.001) (DOCX) [file pone.0185193.s007.docx]

| ALL PATIENTS | Kidney stones: No | Kidney stones: Yes | Total |
| --- | --- | --- | --- |
| Previous hospitalizations in the last 12 months: No | 2132 (97.5%) | 55 (2.5%) | 2187 (100%) |
| Previous hospitalizations in the last 12 months: Yes | 100 (73.5%) | 36 (26.5%) | 136 (100%) |
| Total | 2232 (96.1%) | 91 (3.9%) | 2323 (100%) |

**Table S6:** kidney stones and previous hospitalization in the last 12 months in all patients

OR: 13.95 (95% CI: 8.76 – 22.23; p < 0.001)

Subgroup analysis; CD patients: OR 13.33 (95% CI: 7.54 – 23.56; p < 0.001); UC patients:

OR 14.44 (95% CI: 6.41 – 32.53; p < 0.001)
